# Characterization of the Interaction of Known G‐quadruplex Ligands With a Minimal i‐Motif Structure

**DOI:** 10.1002/chem.71320

**Published:** 2026-06-23

**Authors:** Davide Auricchio, Marko Trajkovski, Janez Plavec, Claudia Sissi

**Affiliations:** ^1^ Department of Pharmaceutical and Pharmacological Sciences University of Padova Padova Italy; ^2^ Slovenian NMR Centre National Institute of Chemistry Ljubljana Slovenia

**Keywords:** circular dichroism, i‐motif, NMR, TMPyP4, UV–vis

## Abstract

Peculiar genomic regions are prone to evolve transiently from double‐helix DNA to noncanonical secondary structures in response to physiological stress. Particularly interesting are G‐rich genomic sites that are known to fold into G‐quadruplexes (G4s) while their complementary C‐rich strands can potentially arrange into i‐Motifs (iMs). Indeed, both may interfere with the regulation of biological processes, including the expression of oncogenes. While G4s have been extensively studied as potential therapeutic targets, iMs were long neglected due to the acidic conditions thought necessary for their stability. Nevertheless, their proven existence within cells has prompted interest in dual G4/iM ligands. Although recent studies suggest that G4 ligands modulate iM stability, elucidating the underlying structural interfaces requires more in‐depth investigations. In this work, we employed NMR to screen four common G4 ligands against a resolved minimal intramolecular iM. By combining UV–vis, CD, and NMR, we characterized the interaction with the most promising ligand, TMPyP4. Our findings highlight a specific structural domain as the preferential binding site for TMPyP4, with the ligand–DNA interactions coupled to a stepwise unfolding of the iM. These results provide structural insights for the rational design of ligands that modulate iM stability and related biological outcomes.

## Graphical abstract

The destabilization of a minimal i‐Motif (iM) structure by TMPyP4 is driven by ligand recognition of one terminal iM structural domain.

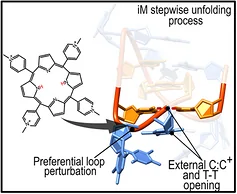

## 1 Introduction

In cells, the formation of transient, noncanonical folded structures along the canonical double‐helix DNA is well‐documented. Their dynamic formation has been related to several biological roles like as telomere maintenance, gene regulation, and cellular homeostasis [1, 2, 3]. Among them, the most widely studied are G‐quadruplex (G4) structures that occur at G‐rich genomic sites [4, 5, 6, 7, 8]. Being significantly enriched at oncogene promoters (e.g., c‐Kit, c‐Myc, HRAS, etc) [2, 9], they have attracted considerable interest as potential targets for therapeutic applications [9, 10]. In most cases, at these genomic sequences, the complementary C‐rich strand holds the sequence requirement to fold into an i‐Motif (iM) structure [11]. However, in the last decades, the biological relevance of iM has been downsized compared to G4, due to the requirement of acidic pH for their folding. Indeed, these structures rely on the formation of C:C^+^ base pairs comprising hemi‐protonated cytosines [11, 12]. For this reason, most drug‐discovery programs focused on ligands for the targeting of G4, without considering the potential iM presence at the complementary strand. However, an increasing line of evidence supports the formation of iM in the cells, leading to growing interest in targeting these structures [13, 14, 15, 16]. In this field, the identification and characterization of selective G4 or iM binders would help to dissect the key cellular mechanisms in which these structures are selectively involved. As well, the potential set‐up of dual G4/iM binders is expected to represent an interesting approach to obtain synergic therapeutic effects [17, 18, 19, 20].

So far, the number of compounds identified as good G4 binders is remarkable. With few exceptions, upon nucleic acids complex formation, they stabilize this tetra‐helical structure. Several of these ligands were also tested for their ability to recognize iM. Nevertheless, among them, no small molecules acting as strong iM stabilizers have been found, while a relevant fraction of them preferentially drives the unfolding of this structure [21, 22]. This output does not elicit their potential relevance for drug development. Indeed, both promotion or reduction of G4 and iM perturbation is expected to affect the overall DNA processing. In addition, cellular models suggested that the formation of G4s and iMs at gene promoters might have opposite effects on the regulation of gene expression in opposite directions [23]. However, a clear understanding of the molecular interaction patterns responsible for the divergent output emerging from G4 versus iM targeting by these small molecules is not available yet.

To provide new insights on this picture, in this work, we selected four DNA ligands previously established as potent G4 binders and endowed with distinct chemical scaffolds (Figure 1b). In detail, BRACO‐19 is an acridine derivative with good selectivity for telomeric G4s and, consistently, capable of blocking their processing by telomerase [24]. PhenDC3 is a bis‐quinoline ligand that displays high affinity and selectivity for both telomeric and promoter G4 structures, efficiently stabilizing quadruplex conformations [25]. We also included Mitoxantrone, an anthraquinone derivative used in clinics as a chemotherapeutic, known as a dsDNA intercalating agent that has been reported to also interact with G4 through stacking interactions [26]. Finally, we considered TMPyP4, a cationic porphyrin that binds G4s through stacking interactions with moderate affinity and limited selectivity versus other DNA foldings [27, 28]. Notably, all these ligands have been reported to interact with iM structures. However, while Braco‐19 and PhenDC3 were confirmed to destabilize iM, for Mitoxantrone and TMPyP4, conflicting evidence reported both stabilizing and destabilizing effects [21, 22, 29, 30, 31]. This variegated output can be largely related to the different structural features of the selected iM used as targets. As an example, it was reported that TMPyP4 did not alter the folding of a tetrameric iM lacking connecting loops [31]. Thus, consistent structural insights into the iM recognition by these ligands are largely missing.

**FIGURE 1:**
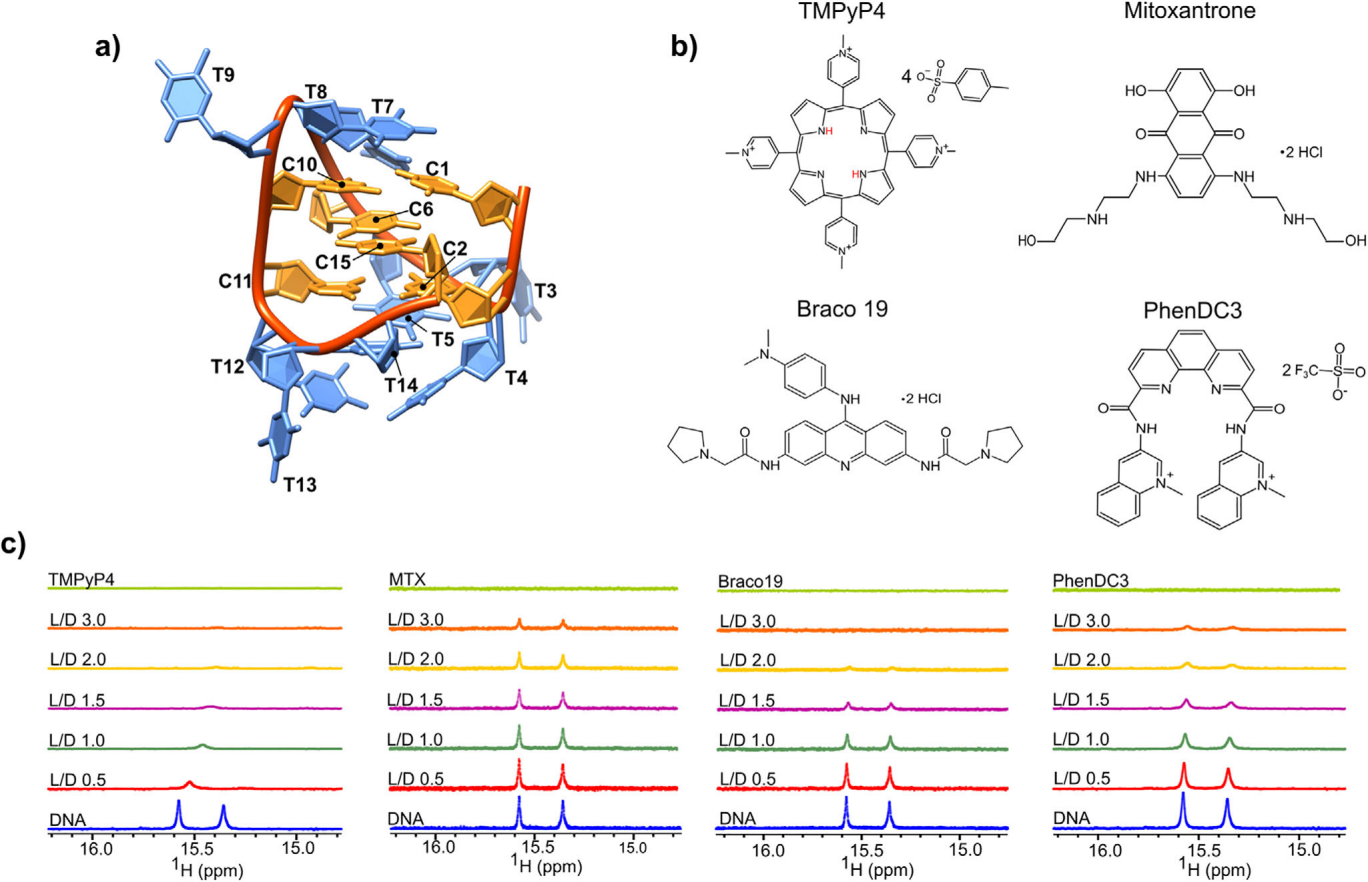
iM and ligands used in this study. (a) NMR‐based solution‐state structure of iM adopted by C21T3 (PDB 8PMB). (b) Chemical structure of the G4‐ligands used in this study. (c) Imino region of the ^1^H NMR spectra of C21T3 acquired in 90%/10% ^1^H_2_O/^2^H_2_O, at 0.2 mM DNA concentration, 25 mM sodium phosphate, pH 5.5, 0°C, at ligand:C21T3 ratio (L/D) ranging from 0 to 3.

Based on these premises, here we used as a drug target the d(CCTTTCTTTCCTTTC)‐3’ sequence, named C21T3, which we previously reported to fold into an iM in acidic conditions (Figure 1a) [32]. The structure of this iM has been solved by NMR (PDB: 8PMB): it comprises three intercalated C:C^+^ base pairs (C1:C10, C6:C15, and C2:C11) connected by three thymine loops. While all the bases of the central loop in the major groove are exposed to the solvent, T5 and T14, from the first and third loop, respectively, are base‐paired and stacked onto C2:C11. Consistently, it represents the simplest model enclosing all the features of an intramolecular iM, thus making it a good working model to investigate how G4 binders can interact with this non‐canonical secondary DNA arrangement. Moreover, having only three C:C^+^ base pairs, this structure is characterized by low thermodynamic stability, making it highly responsive to perturbation in the local microenvironment, like the presence of destabilizing small molecules.

Following our previous experience on its structural characterization, we applied NMR to preliminarily screen the selected ligands for their ability to recognize this minimal iM model. Subsequently, to characterize the DNA binding to the most promising ligand, TMPyP4, we combined NMR, UV absorption, and chiroptical analyses. This allowed us to identify the iM structural elements directly involved in ligand recognition, thus providing useful information for future rational design of selective iM ligands, dual G4/iM binder as well.

## 2 Results and Discussion

### 2.1 G4 Ligands Destabilize C21T3 iM

As introduced, the iM structure adopted by C21T3 has been solved by NMR spectroscopy. Thus, we use this experimental approach to screen its potential interaction with selected G4 ligands. In this regard, we gradually increased ligand concentration in an aqueous solution of C21T3 over a 0 to 3 ligand/C21T3 ratio (L/D) and monitored the corresponding changes in ^1^H NMR spectra. Along these titrations, we focused on the imino ^1^H NMR spectral region where two signals corresponding to the intercalated C2:C11 and C6:C15 pairs are detected around ẟ 15.5 ppm. Notably, the imino ^1^H NMR signal for the C1:C10 base pair is not resolved under any condition due to exposure of the corresponding structural element to the bulk solvent.

Upon addition of any of the selected ligands, ^1^H NMR spectra changes included gradual broadening of imino signals (Figure 1c). These results indicated that selected ligands destabilized C:C^+^ base pairs, leading to the unfolding of the iM structure. Although with slightly different efficiencies, the addition of Braco19, PhenDC3, and Mitoxantrone to C21T3 caused a gradual reduction in the intensities of both imino signals, without notable changes in their chemical shifts. Conversely, TMPyP4 induced marked chemical shift perturbations of both imino signals, C2:C11 and C6:C15, which, in addition, were unevenly broadened, thus suggesting specific destabilizing effects, possibly related to preferential ligand‐DNA binding mode(s). These results warranted a detailed structural characterization of the TMPyP4/C21T3 complex.

### 2.2 UV–Visible and CD Evaluation of TMPyP4/C21T3 Complex Formation

Porphyrins are well‐known efficient DNA ligands, but their binding mode is quite complex since it depends on their structure, on the nucleic acid arrangement, and/or the stoichiometric ratio. As an example, in the presence of dsDNA, TMPyP4 can intercalate between adjacent base pairs or fit within the grooves [33, 34, 35] while, when the target is a G4, stacking on the external tetrads (end‐stacking) is the main interaction mode associated with the stabilization of the folded nucleic acid [36, 37, 38, 39, 40, 41, 42, 43, 44, 45, 46, 47, 48, 49]. However, at high L/D (up to 4/1), additional different binding modes have been observed [46].

These distinct interaction modes have been correlated to unique spectroscopic fingerprints of this ligand. The variation in the intensity and maximal wavelength of the Soret band, centered at 424 nm for the free TMPyP4, along with the profile of the induced CD spectra (iCD) emerging in the same wavelength range upon nucleic acid binding, is indicative of its binding mode with DNA [36, 37, 40, 41, 42, 43, 44, 45]. Typically, large bathochromic shifts (Δ*λ* > 15 nm) and hypochromic effects (H% > 35%) are associated with TMPyP4 intercalation between adjacent base pairs, whereas external groove binding is generally related to smaller Δ*λ* and H% values. On the other side, while positive iCD signals have been related to external binding, a negative chiroptical band is compatible with an intercalative binding mode. Thus, spectroscopic analysis offers a valuable complementary approach for investigating the interaction between TMPyP4 and DNA secondary structures.

Here, we acquired the UV–vis spectra of TMPyP4 upon addition of C21T3 at 0°C in the 300–500 nm wavelength range (Figure 2a). At saturation, the recorded spectrum exhibited a redshift of the maximum adsorption from 424 to 435 nm (Δ*λ* = 11 nm) and a hypocromicity (%H) of about ~ 56% with reference to the one acquired in the absence of DNA. While the observed %H value was in the range of those associated with an intercalative binding mode (> 35.0%), the Δ λ was slightly smaller (> 15 nm). However, these reference values were derived from porphyrin intercalation within long dsDNA constructs, where the end‐stacking mechanism was not significant [43]. Conversely, similar Δ*λ* and %H values were reported for the interaction of TMPyP4 with several G4 and iM structures, where both end‐stacking and/or external binding mode can occur [31, 36, 39, 41, 42]. Additionally, along the titration, we observed an isosbestic point at 430 nm up to L/D = 1, consistent with the distribution of the ligand between a free and a bound state. However, at higher DNA concentration, the isosbestic point was not conserved, and the absorbance at 435 nm increased, thus suggesting the occupancy of additional binding site(s) where the chromophore is hosted with a different orientation. Consistently, by plotting the porphyrin absorbance as a function of DNA concentration both at 424 and 435 nm, we dissected two different binding events. While within a DNA concentration range up to a 1:1 molar ratio, an efficient binding event emerged at both wavelengths (EC_50_ ≈ 0.15 ± 0.06 µM), the signal at 435 nm revealed the formation of a second complex that required higher iM concentration to reach saturation (Figure 2b). Overall, UV–vis data analyses suggested that the interaction between TMPyP4 and C21T3 occurred at multiple binding sites through an end‐stacking or an external binding mechanism. However, it was not possible to discriminate against the main mechanism of interaction between these distinct binding events.

**FIGURE 2:**
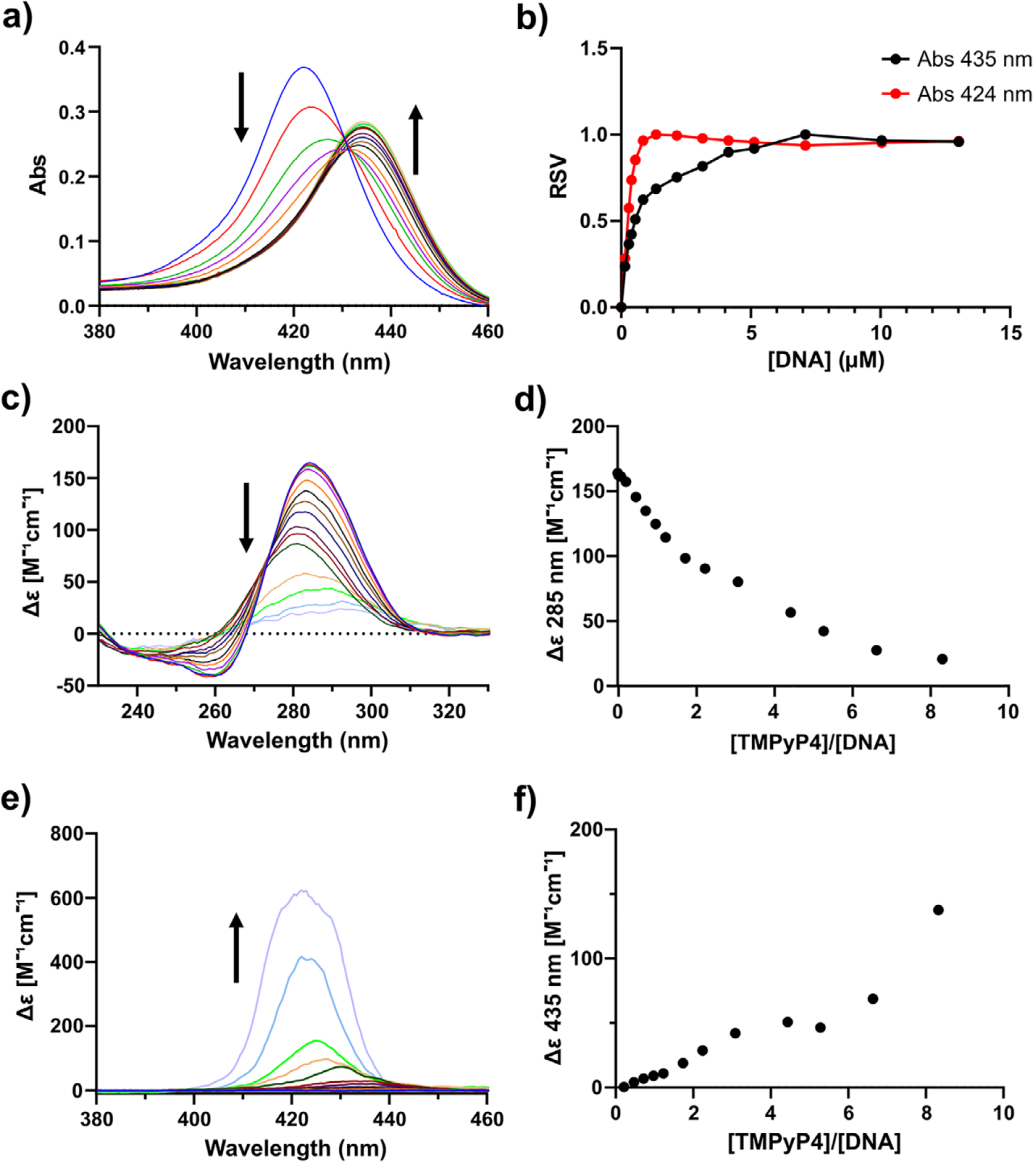
Variation of TMPyP4 absorption and CD spectra upon TMPyP4/C21T3 complex formation. (a) Soret absorption band of 1.6 µM TMPyP4 in 25 mM sodium phosphate, pH 5.5, upon increasing C21T3 concentration and (b) corresponding plot of the Relative Variation of the Signal (RSV) recorded at 435 nm (black line) and at 424 nm (red line). (c) CD spectra of 4 µM C21T3 in 25 mM sodium phosphate, pH 5.5, 0°C upon increasing TMPyP4 concentration and (d) corresponding plot of the CD signal recorded at 285 nm as a function of TMPyP4/DNA molar ratio. (e) ICD in the Vis range acquired upon addition of TMPyP4 to 4 µM C21T3 in 25 mM sodium phosphate, pH 5.5, 0°C, and (f) corresponding plot of the CD signal recorded at 434 nm as a function of the TMPyP4/DNA molar ratio.

To better discriminate between these distinct binding modes, we acquired the CD spectra of C21T3 at increasing concentrations of TMPyP4 (Figure 2c–f). In the absence of the ligand, the positive chiroptical contribution at 285 nm was consistent with the folding of the DNA into an iM. However, the addition of TMPyP4 caused a progressive reduction of this signal, pointing to iM destabilization (Figure 2d). This process was associated with an isosbestic point at 270 nm that was lost at higher TMPyP4 concentration (L/D > 3:1) when the DNA chiroptical contribution remarkably dropped. This suggested the presence of a second ligand binding event involving the unfolded DNA. Consistently, in the visible range, the CD spectra at L/D < 3 revealed the appearance of a positive induced CD (iCD) band centered at 435 nm that, (Figure 2e) according to literature data, suggested an external binding of TMPyP4 to iM rather than its intercalation between adjacent C:C^+^ base pairs [50]. At L/D > 3:1, this positive signal was shifted at shorter wavelengths (up to 424 nm) and showed Δ*ε* value higher than 100 M^−1^ cm^−1^, which is indicative of TMPyP4 self‐stacking (Figure 2e). By normalizing this signal to the porphyrin concentrations, it was possible to observe that this second process reached saturation at L/D close to 10 with an IC_50_ in the mid‐micromolar range (Figure S1).

A direct comparison of the CD and absorbance datasets is not straightforward since they explored different L/D ratios. However, by combining both information, it was possible to address that up to L/D < 3:1, TMPyP4 binds iM preferentially through an end‐stacking or external binding mode. This interaction mode induced a destabilization of the DNA secondary structure; thus, at a higher L/D ratio, the porphyrin binding to DNA appears as a transient state leading to ordered self‐stacking [33]. For proper comparison, these data confirmed that, at the L/D ratio used for our preliminary NMR screening, TMPyP4 did not form self‐aggregates with the unfolded DNA. This finding aligned with our purpose of investigating the structural features of the porphyrin/iM complex.

### 2.3 ^1^H‐NMR Monitored Interactions of C21T3 with TMPyP4

To gain more insights into the TMPyP4 binding mode and to rationalize its destabilizing effect on the iM formed by C21T3, we explored in more detail the NMR data acquired in the range of L/D ratio lower than 3. Notably, the ^1^H NMR spectrum of TMPyP4 alone exhibits a singlet at *ẟ* 9.16 ppm corresponding to the outer pyrrolic protons labeled *β* in Figure 3, which is consistent with the Huckel aromatic nature and symmetry of the molecule. Moreover, *ortho* and *meta* pyridyl protons, marked in Figure 3 as “a” and “b”, are observed as doublet signals at ẟ ^1^H 9.00 and 9.34 ppm respectively, while the N+‐methyl proton labeled “c” resonates at ẟ 4.80 ppm.

**FIGURE 3:**
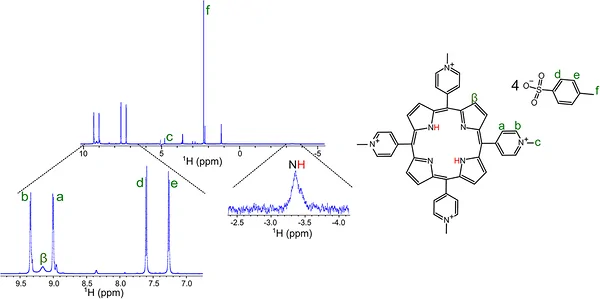
^1^H NMR spectrum of TMPyP4. ^1^H NMR spectrum of 0.2 mM TMPyP4 with expansions on the regions of main interest, recorded in 90%/10% ^1^H_2_O/^2^H_2_O in 25 mM sodium phosphate, pH 5.5, 0°C. On the right side, the structure of the ligand and its counterion, with the labels associated with the main NMR signals, is included.

As far as the exchangeable inner imino protons, it is worth mentioning that although they can be located in *cis* or *trans*, the latter is the most abundant tautomeric form [51, 52, 53]. Interestingly, upon addition of TMPyP4 to C21T3, the signal corresponding to the inner imino protons is shifted upfield (ẟ ^1^H ‐3.35 ppm) due to ring current effects of the macrocyclic core [54, 55, 56]. Finally, the signals labeled as “d”, “e” and “f” refer to protons of the tosylate counter ion, in detail the phenylene protons at ẟ ^1^H 7.60 and ẟ 7.26 ppm, and the methyl proton at ẟ 2.30 ppm.

The incremental addition of TMPyP4 to the iM adopted by C21T3 led to a progressive decrease in imino ^1^H NMR signal intensities (Figure 4a). In detail, the effect was more pronounced for the C2:C11 base pair, whose imino signal broadened to the baseline at an L/D ratio of 0.25, whereas the one for C6:C15 remained discernible up to a ratio of L/D 2.00 (Figure 4b). Crucially, this trend was mirrored by the changes observed in the spectral region for amino protons, with C2 and C11 signals broadening more rapidly than those of C6 and C15 (Figure 4c). These parallel observations across both imino and amino ^1^H NMR signal changes confirm a site‐specific interaction, indicating that TMPyP4 binding preferentially destabilizes the C2:C11 base pair. The site‐specific destabilization is further supported by the ligand concentration‐dependent ^1^H NMR spectral changes observed for the thymine residues. Specifically, the imino signals for T5 and T14, which form a non‐canonical base pair stacked directly over C2:C11, disappear at an L/D ratio of 0.25 (Figure 4d). In contrast, most/several signals corresponding to the T7–T9 loop at the opposite end of the iM remain detectable at ratios as high as 1.50.

**FIGURE 4:**
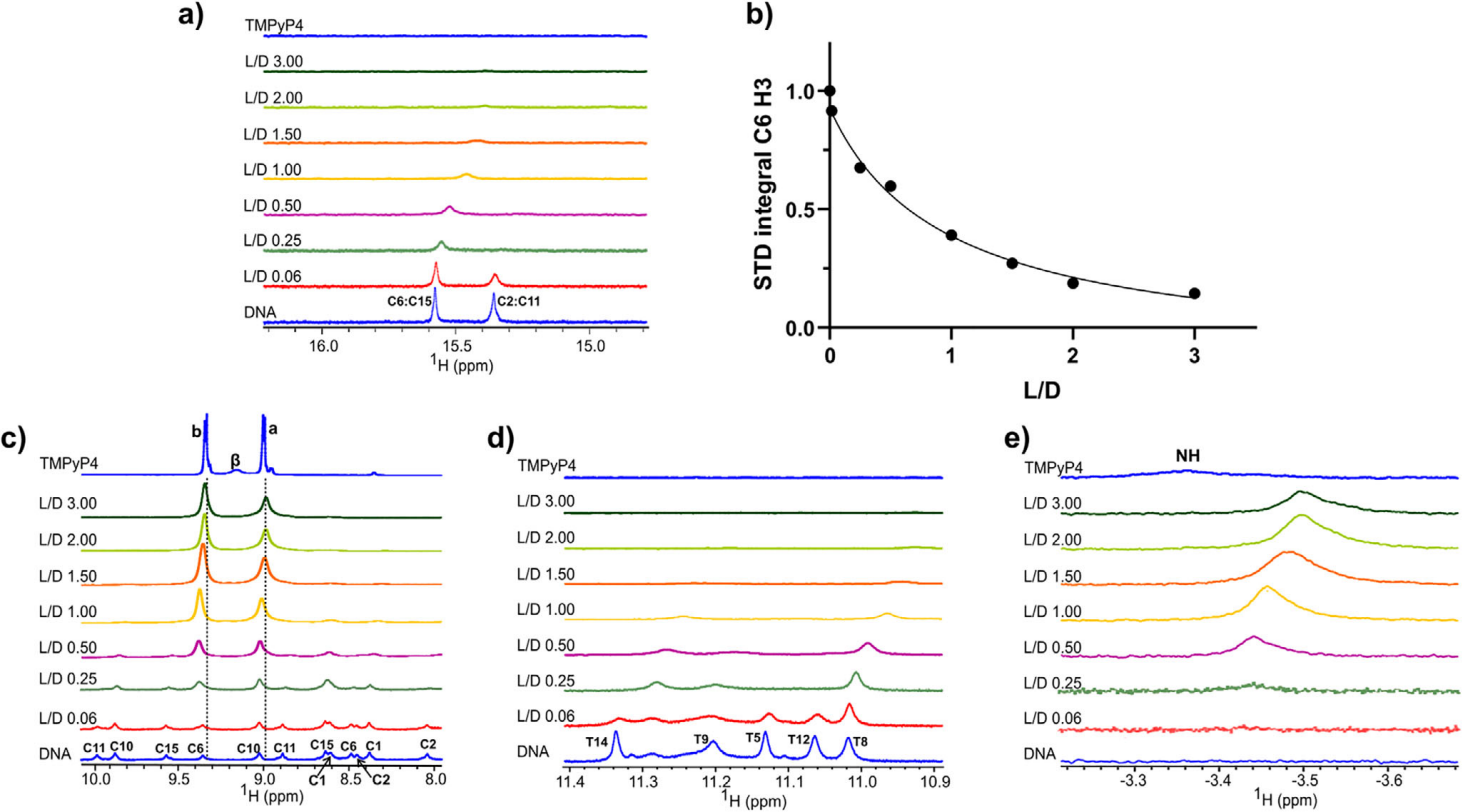
^1^H NMR spectra of C21T3 upon addition of TMPyP4. ^1^H NMR spectral regions showing (a) cytosine imino, (c) cytosine amino, (d) thymine imino, and (e) inner NH ligand of 0.2 mM C21T3 acquired upon titration with TMPyP4 at the reported TMPyP4/C21T3 ratio (L/D up to 3.00). Spectra were recorded in 90%/10% ^1^H_2_O/^2^H_2_O in 25 mM sodium phosphate, pH 5.5, 0°C. (b) Normalized integral value of C6H3 ^1^H NMR signal as a function of L/D ratio. Integral values refer to 0.2 ppm width centered at the maximum of the C6H3 signal.

Analysis of the ^1^H NMR spectral changes reveals that at the lowest ligand concentration, the “a” and “b” pyridyl protons of TMPyP4 (resonating near *ẟ* ^1^H 9.0 and 9.4 ppm; Figure 4c) are shifted slightly downfield relative to the free ligand. As the L/D ratio increases, these signals gradually intensify and shift toward their free‐state chemical shifts, consistent with the saturation of available binding sites and the subsequent accumulation of unbound ligand in solution. Notably, the ligand signals corresponding to the TMPyP4/C21T3 complex are rather broad and could not be resolved from the free‐ligand signals, consistent with low/moderate affinity of binding [57], possibly including equilibrium of different binding interfaces and ligand poses. In contrast, upon titration, the signal for the inner NH protons shifted upfield and narrowed (Figure 4e). This suggests a partial protection from bulk solvent exchange, in line with Chakraborty et al., who reported the intensification of the inner NH signal upon replacement of ^2^H_2_O with ^1^H_2_O [58]. Moreover, its upfield shift in the DNA–ligand complex indicated the insertion of the inner NH protons within an anisotropic shielding environment generated by the adjacent DNA bases.

Collectively, these observations support an off‐center stacking orientation of TMPyP4, as suggested in Figure S2a at the ligand‐terminal C:C^+^ base pair interface.

### 2.4 Insights Into the Structure of the DNA‐TMPyP4 Complex

In contrast to the ^1^H NMR chemical shift variations observed for C21T3 and TMPyP4, the tosylate counter ion signals remained unchanged across the titration range (Figure S2b). Specifically, at both the lowest (L/D 0.5) and highest (L/D 3.0) ratios studied, the signals for “d”,“e”, and “f” protons of tosylate counter ions were consistently observed 0.1 ppm downfield relative to the reference “free ligand” spectrum. Since the tosylate moiety might potentially interact with nucleobases or otherwise mediate interactions between TMPyP4 and C21T3, we applied an ultrafiltration procedure to separate the DNA complex from potentially loosely bound smaller counterions. The ^1^H NMR spectra acquired before and after ultrafiltration were nearly identical, with the notable exception of tosylate signals, which were fully removed. Interestingly, no changes in ^1^H NMR chemical shifts were observed for porphyrin signals, which, however, appeared more resolved in spectra acquired after ultrafiltration (Figure S3). Taken together, the results indicate that removal of tosylate reduced the dynamics of DNA/ligand binding. In view of these results, further structural assessment of TMPyP4 interactions with C21T3 was performed by using ultra‐filtrated samples.

A comparison of the ^1^H NMR spectra of C21T3 alone and in the presence of 0.25 equivalents of TMPyP4 (at 0°C) reveals a lower iM to unfolded strand ratio upon ligand addition, consistent with the destabilization of the DNA secondary structure. Furthermore, ^1^H NMR analysis over the 0°C–15°C range (Figure S4) highlights complex structural dynamics. Specifically, increasing the temperature promotes iM unfolding while simultaneously attenuating the destabilizing effect of the ligand on the iM structure. Notably, CD melting experiments performed in parallel with the NMR analysis confirm that ligand binding is accompanied by a slight destabilization of the iM, whereby Tm decreases from 17.7°C to 14.1°C upon addition of 0.25 equivalents of TMPyP4 (Figure S5). Further structural characterization was performed at 5°C, as this temperature preserves the iM fold while providing better spectral resolution than 0°C.

A detailed structural assessment was performed by comparative analysis between C21T3 in the absence of TMPyP4 and the sample at an L/D ratio of 0.25. We are aware that a saturated system is not achieved at this sub‐stoichiometric ratio and consequently, the observed ^1^H NMR signals correspond to a weighted average of the equilibria between C21T3 in the iM and alternative forms, as well as their respective complexes with TMPyP4. This approach was selected as an optimized compromise, as severe line broadening and limited spectral resolution precluded detailed structural characterization at L/D ratios exceeding 0.25. In detail, inspection of 2D NOESY spectra enabled assignment of the ^1^H NMR chemical shift corresponding to DNA alone (Table S1) and upon TMPyP4 binding (Table S2). The ligand‐induced ^1^H NMR chemical shift perturbations appear slightly larger for the sugar H1’/H2’ (Figure 5a) than nucleobase H6/H5/Me (Figure 5b) protons, which arguably may indicate that TMPyP4 binding is driven by electrostatic rather than stacking interaction.

**FIGURE 5:**
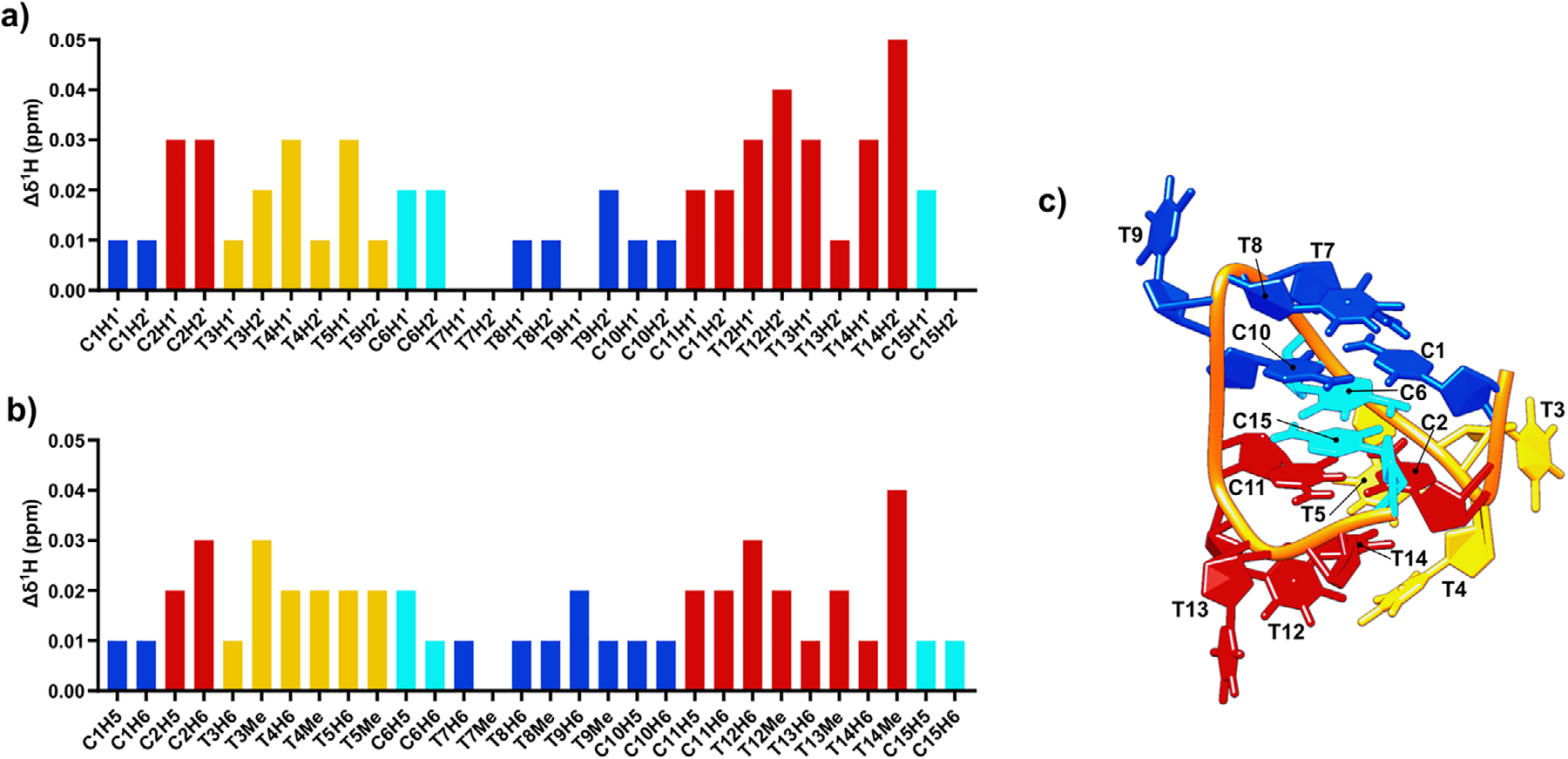
^1^H NMR chemical shift perturbation of C21T3 induced upon addition of TMPyP4 at L/D 0.25. (a) Sugar (protons H1′ and H2′/H2′′) and (b) nucleobases (H5 and H6 for cytosines and H6 and Me for thymines). Δ*ẟ* ^1^H values correspond to the changes in ^1^H NMR chemical shifts after addition of the ligand. For each structural domain (in detail: C1:C10, C2:C11, and C6:C15, base pairs and T3‐T5, T7‐T9, and T12‐T15 loops) the values reported in panel (a) and (b) have been averaged (Δ*ẟ* ^1^H_avg_) and colored according to the resulting values: red (Δ*ẟ* ^1^H_avg_ > 0.09 ppm), orange (Δẟ^1^H_avg_ within 0.06–0.09 ppm), light blue (Δẟ^1^H_avg_ within 0.04–0.06 ppm), and blue (Δẟ^1^H_avg_ < 0.04 ppm). (c) Graphical representation of C21T3 iM structure, where the residues have been colored accordingly.

The most pronounced chemical shift perturbations were observed for residues T14, T12, and C2, followed by C11. In view that in this iM the C2:C11 base pair is capped by a noncanonical T14:T5 base pair, the larger Δ*ẟ* ^1^H values observed for the T12–T14 loop in comparison to the T3–T5 suggest the T12–T14 loop as a preferential binding site. Specifically, the binding appears to be guided by electrostatic interactions between the cationic pyridyl arms and the negatively charged sugar–phosphate backbone, together with stacking between the pyrrolic rings and nucleobases [30]. These findings are consistent with an off‐center binding mode, as depicted in Figure S2. Notably, in view that the ^1^H NMR signals used to assess the chemical shift perturbations represent weighted averages of multiple species in equilibria, we performed a comparative analysis of the NOESY spectra of C21T3 alone and in the presence of TMPyP4. Moreover, this approach afforded enhanced spectral resolution, enabling the verification of ^1^H NMR signal assignments. Furthermore, inspection of NOESY cross‐peaks revealed relative differences in proton–proton distances between the ligand‐free and ligand‐bound DNA, altogether providing a robust dataset to complement the results inferred from the ^1^H NMR chemical shift perturbations.

Analysis of the ligand‐induced changes in NOESY spectra reveals a significant attenuation of inter‐residue cross‐peaks. In particular, the imino–imino cross‐peak corresponding to the noncanonical T5:T14 base pair appears markedly reduced upon addition of TMPyP4, consistent with the destabilizing effect of TMPyP4 on the iM structure (Figure 6). In fact, the NOE interactions between T14H2′–C2H1′ and T5H2′–C11H1′ are observed only before, but not after, the addition of TMPyP4 (Figure S6), which suggests that the ligand binding results in simultaneous opening of T5:T14 and C2:C11 base pairs. The coupled structural changes point to synchronized destabilization of the iM. Interestingly, the reduction in inter‐residual NOESY cross‐peaks is more pronounced for the T12–T14 than the T3–T5 loop (Figure S7), thus suggesting that destabilization of the iM structure originates from preferential TMPyP4 binding to residues in the C11‐T12‐T13‐T14 region.

**FIGURE 6:**
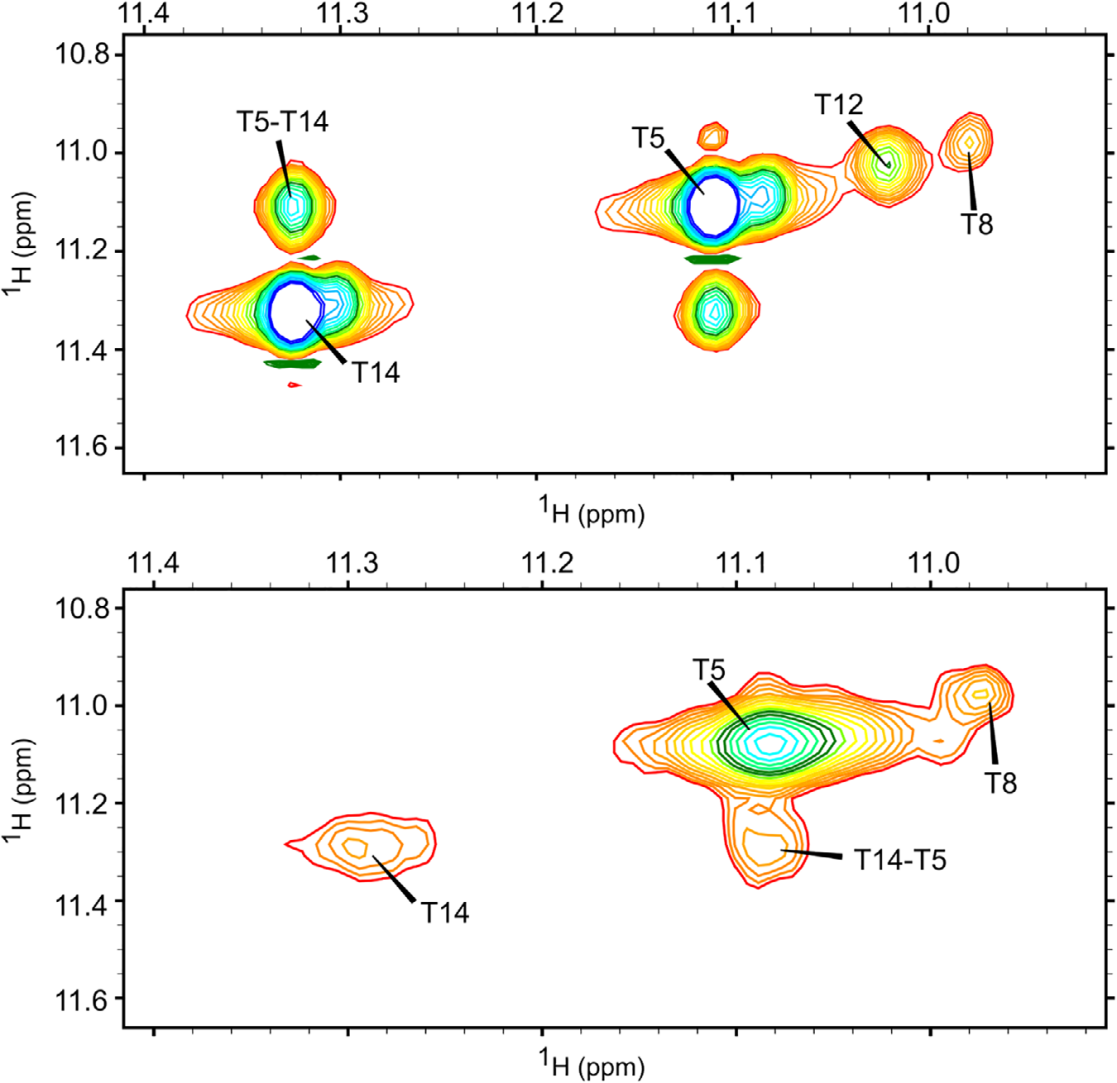
Opening of T14:T5 base pair upon TMPyP4 binding. 2D NOESY (*τ*_m_ 200 ms) spectral region showing characteristic thymine imino–imino cross‐peaks. The spectrum was acquired of 0.24 mM C21T3(upper panel) and TMPyP4/C21T3 complex at L/D 0.25 molar (bottom panel), in 90%/10% ^1^H_2_O/^2^H_2_O, 25 mM sodium phosphate, pH 5.5, 5°C.

The persistence of C15H42‐C11H5 and C11H42‐C15H5 as well as of C2H42‐C6H5 NOE cross‐peaks in the presence of the ligand (Figure S8) is consistent with at least partly preserved interactions between C11 and C15 as well as between C2 and C6. This is corroborated by the observed C11H1′–C6H4′ and C2H1′–C15H5′/H5′′ cross‐peaks in the absence as well as in the presence of the ligand (Figure S9). The formation of C1:C10 base pair even upon addition of the ligand is supported by several NOESY cross‐peaks, including C1H2′‐C6H42, C1H2′′‐C6H42, C6H2′′‐C1H42, C10H2′‐C15H42, and C10H2′′‐C15H42, consistent with the proximity of the sugar and amino protons of the adjacent cytosines (Figure S10). The robustness of this iM part is further corroborated by inter‐residual NOE interactions for the T7–T8–T9 loop (Figure S11).

## 3 Conclusion

The literature that focuses on the identification and characterization of G4 ligands is far more extensive than that of iM binders. This can be related to a general iM destabilization observed upon ligand binding [21, 22]. The herein reported NMR screening performed using a minimal iM structure as a target confirmed this behavior for a few selected G4 ligands. Notably, it turned out to be a good working model to efficiently describe from a structural point of view the ligand–iM interaction modes of TMPyP4 and ultimately provide the rationale behind this outcome. For the selected TMPyP4, this was intriguing since, as previously reported for other DNA structures, it can shift among different binding modes in accordance with the L/D ratio.

UV–vis and CD spectroscopy data revealed that for L/D ≤ 3, redshift (Δ*λ*) and hypocromicity (%H) values, and positive iCD supported an end‐stacking and/or external binding mode rather than an intercalation within the C:C^+^ base pairs, consistent with previous works on telomeric iMs [31, 50, 59]. Importantly, the herein studied minimal iM was unfolded upon the addition of TMPyP4 interaction, which is similar to reports focusing on the iM from the Bcl‐2 oncogene [30] and in contrast to studies on tetrameric iM, where folding was preserved upon ligand binding [31]. Thus, it is useful to address the cause of this differening results.

The resolved structure of a tetrameric iM/TMPyP4 complex was obtained by using C‐rich strands with a 5′‐overhang (d(AACCCC)). Here, two ligands are symmetrically located at the opposite sides of the iM core at the interface with the unpaired adenines. In contrast, our NMR data indicated that TMPyP4 binding to C21T3 preferentially affected one iM side, in particular the T12–T14 loop and the adjacent C2:C11 base pair. The ^1^H NMR chemical shift perturbation analysis revealed that at these sites, the sugar protons were more affected than those of the nucleobases. In the studied iM structure, the higher negative charge density was expected to be located in the narrow groove at the C2:C11 base pair side, where two loops are present. Here, the initial electrostatic recognition was further reinforced by stacking interactions between the pyrrolic rings and nearby nucleobases, ultimately influencing the inner NH proton tautomerism of TMPyP4. Indeed, the two loops proximal to the C2:C11 base pairs were perturbed distinctly, suggesting an off‐center binding of TMPyP4 to C21T3 iM. This feature was in line with the previously suggested role of the loops in guiding the overall intramolecular iM stability [30]. Indeed, in our model, the loss of the T14:T5 capping element is synchronized with the opening of the C2:C11 base pair and reduced iM thermal stability.

Notably, the perturbation of C2:C11 and T14:T5 base pairs led to differential long‐range effects. Indeed, the NMR data indicate that the addition of TMPyP4 increases the flexibility of the T12–T14 loop, while the T3–T4–T5 loop remains in proximity to C2 in the major groove. At the same time, the conformation of C1:C10 and, to a lesser extent, the C6:C15 base pairs and T7–T9 loop remains largely unaltered. This pointed to the absence of porphyrin at this domain, at a low L/D ratio. This binding clearly describes a stepwise iM destabilization, where local disruption of loop and base pair interactions propagated toward the iM core, ultimately leading to a DNA‐TMPyP4 complex that does not include iM.

It is worth underlining that, although along the human genome up to almost 700 sites corresponding to the herein used DNA sequence can be counted (human reference genome: Hg38), the associated iM structure is not expected to play relevant roles *in cells* due to its limited intrinsic stability [32]. However, here we confirmed how this minimal model can be proficiently used to derive valuable insights into the recognition of precise structural elements, a valuable knowledge to support the design of selective iM ligands. Finally, since the complementary G‐rich‐strand also does not fold into a G4, in the future it will be interesting to confirm our conclusions on more complex models to proficiently translate our findings to the design of dual G4/iM binders.

## Conflicts of Interest

The authors declare no conflicts of interest.

## Supporting information




**Supporting File**: chem71320‐sup‐0001‐SuppMat.docx

## Data Availability

The data that support the findings of this study are available in the Supporting Information of this article.
