# Supplementary material for: Characterization of the Interaction of Known G‐quadruplex Ligands With a Minimal i‐Motif Structure
Source: Chemistry. 2026 Jun 23;32(37):e71320. doi: 10.1002/chem.71320 (PMC13628523; doi:10.1002/chem.71320)
Supplement: Supplementary file 1 [file CHEM-32-e71320-s001.docx]

**Supporting Information**

Characterization of the interaction of known G-quadruplex ligands with a minimal i-Motif structure

Davide Auricchio^[a]^, Marko Trajkovski^[b]^, Janez Plavec^[b]*^ and Claudia Sissi^[a]*^

[a]	Dr. Davide Auricchio, Prof. Claudia Sissi
Department of Pharmaceutical and Pharmacological Sciences,
University of Padova
Via Marzolo 5, 35131 Padova, Italy
E-mail: [claudia.sissi@unipd.it](mailto:claudia.sissi@unipd.it)

[b]	Dr. Marko Trajkovski, Prof. Janez Plavec
Slovenian NMR Centre,
National Institute of Chemistry,
Hajdrihova 19, 1000 Ljubljana
E-mail: janez.plavec@ki.si

**Table of Contents**

MATERIAL AND METHODS……………………………………………………………………………………………3

SAMPLE PREPARATION……………………………………………………………………………………..3

NMR SPECTROSCOPY……...……………………………………………………………………………….3

UV-VIS TITRATION…………………………………….……………………………………………………...3

CD SPECTROSCOPY TITRATION..…………………………………………………………………………3

SUPPLEMENTARY FIGURES…………………………………………………………………………………………4

SUPPLEMENTARY TABLES………….………………………………………………………………………………10

**Materials and Methods**

**Sample preparation**

The non-labelled and the residue-specific partially (8 %) ^15^N-isotopically labelled oligonucleotide C21T3 (d(CCTTTCTTTCCTTTC)) was synthesized on K&A Laborgeraete GbR DNA/RNA Synthesizer H-8 via standard phosphoramidite chemistry. Deprotection was achieved by overnight incubation in aqueous ammonia at 55 °C. Purification and desalting were achieved with the use of Amicon Ultra-15 Centrifugal Filter Units.

The ligands PhenDC3 (Mw =848.75 g/mol, Ɛ_370_ =62400 M^-1^ cm^-1^), Braco 19 (Mw =593.76 g/mol, Ɛ_363_=62400 M^-1^ cm^-1^), Mitoxantrone (Mw =517.40 g/mol, Ɛ_682_ = 8360 M^-1^ cm^-1^) and TMPyP4 (Mw =1363.60 g/mol, Ɛ_424_ = 226000 M^-1^ cm^-1^) were purchased from Sigma-Aldrich. Ligand stock solutions were prepared at 24 mM in MQ water.

**NMR spectroscopy**

^1^H NMR samples were prepared by dissolving C21T3 in 25 mM sodium phosphate, pH 5.5, at a final concentration of 0.2 mM in 90%/10% H_2_O/D_2_O. Titration was performed by incremental addition of ligand stock solutions up to a ligand/DNA (L/D) ratio of 3. NMR spectra were recorded on 600 and 800 MHz spectrometers equipped with HCN cryogenic probes within 0-15 °C.

2D NMR experiments of free DNA and TMPyP4/DNA complex (L/D 0.25), were performed at 5 °C. Tosylate counter ions were removed from the L/D 0.25 complex by five ultrafiltration steps using Amicon tubes with a 1 kDa MWCO. At each step, the sample was washed with fresh buffer. At the end of the procedure, the reduction of ionic strength deriving from counterion removal was compensated by the addition of 12 mM NaCl. NOESY spectra of free DNA and L/D 0.25 complex were acquired at a 200-ms mixing time (τ_m_). NMR spectra were processed and analyzed by using TopSpin (Bruker) and Sparky (UCSF) software.

**UV-Vis titration**

UV-Vis spectra were acquired on a Jasco-V710 spectropolarimeter equipped with a Peltier accessory for temperature control at 0 °C, using a quartz cuvette with 1 cm pathlength in the 300-500 nm range. TMPyP4 was prepared at 1.6 μM in 25 mM sodium phosphate, pH 5.5. A solution of C21T3 (325 μM, strand concentration) previously annealed in the same buffer was progressively added to the TMPyP4 solution. UV-Vis titrations ended when the porphyrin absorption spectrum did not change upon three successive additions of C21T3. The percentage of hypochromic variation (%H) of the porphyrin Soret band at 424 nm was calculated using the following formula: %H = [(ε_f_− *ε*_b_)/*ε*_f_] × 100, where *ε*_b_ = *A*_b_/*C*_b_. *ε*_f_ and *ε*_b_ referred to the extinction coefficient of the free (226000 M^-1^ cm^-1^) and bound porphyrin (99100 M^-1^ cm^-1^), respectively.

At any titration point, the Relative Signal Variation (RSV) was calculated at 424 nm and 435 nm using the equation, RSV = (A_f_ − A)/(A_f_ − A^b^), where A_f_ and A_b_ were the absorbance of the free and fully bound TMPyP4. The concentrations of free (C_f_) and bound TMPyP4 (C_b_) were derived using C_f_ = C (1 − α) and C_b_ = C − C_f_, respectively. The variation of the TMPyP4 UV absorption at a fixed wavelength was fitted to derive the DNA concentration required to produce 50 % of the maximal absorbance change (EC_50_).

**CD spectroscopy titration**

CD spectra were acquired using a Jasco J-815 spectropolarimeter equipped with a Peltier accessory for temperature control, using a quartz cuvette with 1 cm path length. C21T3 solutions were prepared in 25 mM sodium phosphate, pH 5.5, at 4.0 μM oligo concentration. CD spectra were recorded at 0 °C from 230 to 500 nm, with increasing TMPyP4 concentration (from 0.025 to 11.7 L/D ratio). Each spectrum represents the average of 2 scans acquired with the following parameters: scanning speed 100 nm/min; band width of 2 nm; data interval of 0.5 nm; response of 2 s. CD melting experiments were carried out in the 0-60 °C range at 0.33 °C/min scan rate at 4.0 μM concentration for C21T3 and at 40.0 μM concentration for C21T3 with 10 μM TMPyP4 (L/D 0.25). After subtraction of the buffer baseline, CD spectra were normalized according to the following formula: Δε= θ / (C*l*32980), in units [M^−1^cm^−1^], where θ is the acquired signal in millidegrees, C is the analyte molar concentration and l is the path length of the cell (cm).

**Supplementary Figures**

**Figure S1.** a) Induced CD spectra of TMPyP4 and b) variation of the signal recorded at 424 nm (right panel) upon porphyrin addition to a solution of 4 uM C21T3 in 25 mM sodium phosphate, pH 5.5, 0 °C.

**Figure S2**. **a)** Model of TMPyP4 trans tautomeric forms upon binding to the i-Motif. The upper panel describes the equilibrium between two degenerate trans tautomeric forms where light blue dots have the same chemical shift for imino inner protons. The bottom panel shows the asymmetry induced by DNA binding to TMPyP4. The green circle underlines the TMPyP4 NH inner proton that changed the chemical shift upon C21T3 binding. **b)** Perturbation of ^1^H chemical shift of TMPyP4 upon binding to C21T3 in 25 mM sodium phosphate, pH 5.5, 0 °C. The reported Δδ were derived as follow: δ_(bound ligand)_ – δ_(free ligand)_. Protons are named according to Figure 3. The signals of protons a (blue line) and c (green line) significantly overlapped.

**Figure S3**. **a)** Counter ion and **b)** TMPyP4 protons region of the ^1^H NMR spectra of TMPyP4, TMPyP4/C21T3 complex at L/D 0.25 molar ratio before and after filtration procedure recorded in 90%/10% ^1^H_2_O/^2^H_2_O, 25 mM sodium phosphate, pH 5.5, 5 °C. Black arrows point to the disappearance of the counter ion signals at ẟ 7.68 ppm, ẟ 7.38 ppm and ẟ 2.40 ppm

**Figure S4**. Amino region of the ^1^H NMR spectra of TMPyP4/C21T3 complex at L/D 0.25 molar ratio acquired from 0 °C to 15 °C. Temperatures are shown on the left of the corresponding spectrum. The spectra were recorded in 90%/10% ^1^H_2_O/^2^H_2_O at 0.4 mM DNA, 0.1 mM TMPyP4, 25 mM sodium phosphate buffer (pH 5.5). Red stars highlight the resolution of the overlapped peaks, with the appearance of C6H41 and C10H42 signals.

**Figure S5.** a) Comparison of CD spectra of 4 μM C21T3 (blue line) and 40 μM C21T3 with 10 μM TMPyP4 (L/D 0.25) (red line) in 25 mM sodium phosphate, pH 5.5, 0 °C. b) Variation of CD signal at 285 nm as a function of the temperature for 4 μM C21T3 (blue line) and 40 μM C21T3 with 10 μM TMPyP4 (L/D 0.25) (red line) in 25 mM sodium phosphate, pH 5.5.

**Figure S6**. 2D NOESY (τ_m_ 200 ms) spectral region showing characteristic anomeric-aromatic protons cross-peaks. The spectrum was acquired of 0.24 mM C21T3 (left panel) and TMPyP4/C21T3 complex at L/D 0.25 molar (right panel), in 90%/10% ^1^H_2_O/^2^H_2_O, 25 mM sodium phosphate, pH 5.5, 5 °C.

**Figure S7**. 2D NOESY (τ_m_ 200 ms) spectral region showing characteristic anomeric-anomeric protons cross-peaks. The spectrum was acquired of 0.24 mM C21T3 (upper panel) and TMPyP4/C21T3 complex at L/D 0.25 molar (bottom panel), in 90%/10% ^1^H_2_O/^2^H_2_O, 25 mM sodium phosphate, pH 5.5, 5 °C.

**Figure S8**. 2D NOESY (τ_m_ 200 ms) spectral region showing characteristic amino-aromatic protons cross-peaks. The spectrum was acquired of 0.24 mM C21T3 (left panel) and TMPyP4/C21T3 complex at L/D 0.25 molar (right panel), in 90%/10% ^1^H_2_O/^2^H_2_O, 25 mM sodium phosphate, pH 5.5, 5 °C.

**Figure S9**. 2D NOESY (τ_m_ 200 ms) spectral region showing characteristic anomeric-anomeric protons cross-peaks. The spectrum was acquired of 0.24 mM C21T3 (upper panel) and TMPyP4/C21T3 complex at L/D 0.25 molar (bottom panel), in 90%/10% ^1^H_2_O/^2^H_2_O, 25 mM sodium phosphate, pH 5.5, 5 °C.

**Figure S10**. 2D NOESY (τ_m_ 200 ms) spectral region showing characteristic amino-anomeric protons cross-peaks. The spectrum was acquired of 0.24 mM C21T3 (left panel) and TMPyP4/C21T3 complex at L/D 0.25 molar (right panel), in 90%/10% ^1^H_2_O/^2^H_2_O, 25 mM sodium phosphate, pH 5.5, 5 °C.

**Figure S11**. 2D NOESY (τ_m_ 200 ms) spectral region showing characteristic methyl-anomeric protons cross-peaks. The spectrum was acquired of 0.24 mM C21T3 (left panel) and TMPyP4/C21T3 complex at L/D 0.25 molar (right panel), in 90%/10% ^1^H_2_O/^2^H_2_O, 25 mM sodium phosphate, pH 5.5, 5 °C.

**Supplementary Tables**

**Table S1.** ^1^H NMR assignment for C21T3. The chemical shifts, reported in ppm, were derived from spectra recorded at 0.2 mM C21T3 in 90%/10% ^1^H2O/^2^H2O, 25 mM sodium phosphate buffer, pH 5.5 at 5 °C.

| Residue/proton | H1' | H2'/H2'' | H3' | H5/Me | H6 |
| --- | --- | --- | --- | --- | --- |
| C1 | 6.26 | 2.06/2.53 | 4.86 | 6.02 | 7.76 |
| C2 | 6.56 | 2.36/2.54 | 4.80 | 5.86 | 7.78 |
| T3 | 6.45 | 2.08/2.56 | 4.81 | 1.99 | 7.94 |
| T4 | 6.43 | 2.37/2.55 | 4.86 | 1.95 | 7.85 |
| T5 | 6.00 | 1.67/2.75 | 4.89 | 1.91 | 7.87 |
| C6 | 6.29 | 1.82/2.67 | 4.89 | 6.05 | 7.74 |
| T7 | 6.26 | 2.28/2.50 | 4.80 | 1.99 | 7.79 |
| T8 | 6.00 | 2.06/2.32 | 4.76 | 1.80 | 7.51 |
| T9 | 6.45 | 2.24/2.52 | 4.56 | 1.90 | 7.78 |
| C10 | 6.44 | 2.26/2.79 | 4.88 | 6.30 | 7.94 |
| C11 | 6.46 | 2.27/2.63 | 5.03 | 6.09 | 7.77 |
| T12 | 6.25 | 2.07/2.66 | 4.99 | 1.95 | 7.87 |
| T13 | 6.32 | 1.90/2.47 | 4.88 | 1.89 | 7.75 |
| T14 | 5.91 | 2.00/2.53 | 4.93 | 1.79 | 7.62 |
| C15 | 4.48 | 2.34/2.43 | 4.38 | 6.03 | 7.80 |

**Table S2**. ^1^H NMR assignment for the TMPyP4/C21T3 complex at L/D 0.25. The chemical shifts, reported in ppm, were derived from spectra recorded at 0.2 mM C21T3 and 0.05 mM TMPyP4, in 90%/10% ^1^H2O/^2^H2O, 25 mM sodium phosphate buffer, pH 5.5 at 5 °C.

| Residue/proton | H1' | H2'/H2'' | H3' | H5/Me | H6 |
| --- | --- | --- | --- | --- | --- |
| C1 | 6.25 | 2.05/2.52 | 4.84 | 6.01 | 7.75 |
| C2 | 6.53 | 2.33/2.51 | 4.77 | 5.84 | 7.75 |
| T3 | 6.44 | 2.06/2.53 | 4.78 | 1.96 | 7.93 |
| T4 | 6.40 | 2.36/2.55 | 4.86 | 1.93 | 7.83 |
| T5 | 5.97 | 1.66/2.73 | 4.88 | 1.89 | 7.85 |
| C6 | 6.27 | 1.80/2.66 | 4.89 | 6.03 | 7.73 |
| T7 | 6.26 | 2.28/2.49 | 4.79 | 1.99 | 7.78 |
| T8 | 5.99 | 2.05/2.32 | 4.75 | 1.79 | 7.50 |
| T9 | 6.45 | 2.22/2.52 | 4.55 | 1.89 | 7.76 |
| C10 | 6.43 | 2.25/2.77 | 4,86 | 6.29 | 7.93 |
| C11 | 6.44 | 2.25/2.62 | 5.02 | 6.07 | 7.75 |
| T12 | 6.22 | 2.03/2.63 | 4.96 | 1.93 | 7.84 |
| T13 | 6.29 | 1,89/2.45 | 4.86 | 1.87 | 7.74 |
| T14 | 5.88 | 1.95/2.50 | 4.90 | 1.75 | 7.61 |
| C15 | 4.46 | 2.34/2.40 | 4.37 | 6.02 | 7.79 |
